# Gn protein expressed in plants for diagnosis of severe fever with thrombocytopenia syndrome virus

**DOI:** 10.1007/s00253-024-13135-0

**Published:** 2024-04-19

**Authors:** Yu-Chih Chang, Hiroshi Shimoda, Min-chao Jiang, Yau-Heiu Hsu, Ken Maeda, Yumiko Yamada, Wei-Li Hsu

**Affiliations:** 1 Doctoral Program in Microbial Genomics, National Chung Hsing University and Academia Sinica, Taichung, Taiwan; 2 Graduate Institute of Microbiology and Public Health, College of Veterinary Medicine, National Chung Hsing University, Taichung, Taiwan; 3 Laboratory of Veterinary Microbiology, Joint Faculty of Veterinary Medicine, Yamaguchi University, Yamaguchi, Japan; 4 Graduate Institute of Biotechnology, National Chung Hsing University, Taichung, Taiwan; 5 Department of Veterinary Science, National Institute of Infectious Disease, Tokyo, Japan; 6 The iEGG and Animal Biotechnology Center, National Chung Hsing University, Taichung, 402 Taiwan

**Keywords:** Severe fever with thrombocytopenia syndrome virus **(**SFTSV), Glycoprotein N (Gn), *Bamboo mosaic virus*-based vector system, Glycosylation, Secretory signal tags, Seroprevalence

## Abstract

**Abstract:**

Severe fever with thrombocytopenia syndrome virus (SFTSV) causes the highly fatal disease in humans. To facilitate diagnosis, the native form of subunit glycoprotein (Gn), a prime target for potential vaccines and therapies, was produced in *Nicotiana benthamiana* using a Bamboo mosaic virus-based vector system. By fusion with secretory signal tags, SS^Ext^, derived from the extension protein, and the (SP)_10_ motif, the yield of the recombinant Gn (rGn) was remarkably increased to approximately 7 mg/kg infiltrated leaves. Ultimately, an rGn-based ELISA was successfully established for the detection of SFTSV-specific antibodies in serum samples from naturally infected monkeys. As validated with the reference method, the specificity and sensitivity of rGn-ELISA were 94% and 96%, respectively. In conclusion, utilizing well-suited fusion tags facilitates rGn production and purification in substantial quantities while preserving its antigenic properties. The rGn-ELISA, characterized by its commendable sensitivity and specificity could serve as a viable alternative diagnostic method for assessing SFTSV seroprevalence.

**Key points:**

• *SFTSV Gn, fused with secretory signal tags, was expressed by the BaMV-based vector.*

• *The plant fusion tags increased expression levels and eased the purification of rGn.*

• *The rGn-ELISA was established and validated; its specificity and sensitivity > 94%.*

**Supplementary Information:**

The online version contains supplementary material available at 10.1007/s00253-024-13135-0.

## Introduction

Severe fever with thrombocytopenia syndrome (SFTS) represents an emerging tick-borne viral infectious disease. Following its initial human reports in China, the incidence of SFTS cases has spread to neighboring countries, including Japan, South Korea, Vietnam, Thailand, Pakistan, Myanmar, and Taiwan (Kim et al. 2013; Lin et al. 2020; Rattanakomol et al. 2022; Takahashi et al. 2014; Tran et al. 2019; Zhang et al. 2013). Ticks are strongly implicated as the primary transmission vectors for SFTSV, while domestic animals are considered potential amplifying hosts. The causative agent is severe fever with thrombocytopenia syndrome virus (SFTSV), named in recognition of the syndrome itself (Zhang et al. 2013), also known as *Dabie bandavirus*, which falls within the genus *Bandavirus* in the *Phenuiviridae* family. It is a negative-strand RNA virus, the genome comprises three segments: L, M, and S. The L segment encodes the RNA-dependent RNA polymerase (RdRp), while the S segment expresses both nucleocapsid proteins (NP) and nonstructural proteins (NSs). The M segment encodes the surface glycoprotein precursor (Gp) that is subsequently cleaved into glycoprotein N (Gn) and glycoprotein C (Gc) by host proteases (Wu et al. 2017). Gn and Gc play pivotal roles in receptor binding and membrane fusion, with Gn being a target protein susceptible to neutralizing antibodies.

Noteworthy, fatality rates of SFTS range from 5 to 30%. The increasing number of cases and the severity of symptoms have prompted the World Health Organization (WHO) to designate SFTSV as a priority pathogen demanding immediate attention (World Health Organization 2018, February 6). Nevertheless, up to now, vaccines or antiviral medications for either the prevention or treatment of SFTSV infection are not available. Hence, diagnosis and surveillance of SFTS are critical for the control of such a deadly infectious disease. For the laboratory diagnosis of SFTSV, nucleic acid amplification-derived methods have proven effective in detecting viral RNA in serum samples (Kuan et al. 2023), particularly during the acute phase of infection, which occurs within the first week of onset (Sun et al. 2012). It is important to note that the viremic period is relatively shorter compared to the duration of antibody production following infection. Therefore, serological methods could be considered ideal for monitoring the infection status to mitigate the risk of false negatives. Previous research has reported that antibodies isolated from a patient who had successfully recovered from SFTSV infection demonstrated reactivity to the envelope Gn glycoprotein of SFTSV. These antibodies ultimately protected against infection, as evidenced by both in vitro and in vivo experiments (Kim et al. 2019). These observations strongly suggest that Gn-specific antibodies exhibit robust reactivity with the virus and may lead to a long-lasting immune response.

In recent years, plants have gained substantial attention as a promising expression system for the cost-effective production of biologics on a large scale. This trend is expected to endure, driven by the increasing demand for pharmaceuticals (Chen et al. 2012). Compared to bacterial expression systems, plants, while potentially yielding lower quantities, possess the inherent capability of post-translational modifications similar to those found in animal cells (Castilho and Steinkellner 2012). Furthermore, they offer advantages such as native protein folding and the absence of inclusion bodies, as well as extremely low levels of endotoxins, which are the well-known limitation of prokaryotic systems (such as *Escherichia coli*), rendering them well-suited for the expression of intricate proteins such as antibodies and Fc-antigen fusion proteins (Kim et al. 2018), as well as viral antigens (Huang et al. 2008; Kalthoff et al. 2010), and human interferon (Islam et al. 2019; Jiang et al. 2019). Noteworthy, numerous strategies have been devised to elevate yields and enhance the secretion of proteins. A study reported the protocols for high-level production of human KD1 protein with biological activity in *Nicotiana benthamiana* (*N. benthamiana*) via a *Tobacco mosaic virus* (TMV) vector (Williams et al. 2014). Moreover, one previous research has demonstrated that by incorporation of SS^Ext^, the secretory signal derived from the extensin protein of *N. benthamiana*, the yield, and solubility of the recombinant interferon-γ protein were significantly enhanced in a protein expression system based on *Bamboo mosaic virus* (BaMV) (Jiang et al. 2020). Furthermore, it has been reported that heterogeneous human protein (IFN-α2b) when expressed as an arabinogalactan fusion protein by the inclusion of the tandem repeats of “Ser-Pro” motifs from hydroxyproline-O-glycosylated peptides (HypGPs), exhibited a remarkable increase in secreted yields by 350 to 1400-fold compared to the non-glycosylated control. This modification also increased the half-life of IFN-α2b in serum without affecting its bioactivity (Xu et al. 2007).

In addition to the use of signal peptides, Kang et al., reported the recombinant prostate acid phosphatase (PAP) protein expressed as a fusion protein with the Fc domain of immunoglobulin (Ig) A or M expressed in transgenic plants (Kang et al. 2023). The plant-derived recombinant PAP-Fc fusion proteins retained the polymeric shape representing the IgA or IgM and were able to elicit a robust immune response against prostate cancer. Moreover, a vector system called “magnifection” was developed to facilitate the formation of active replicons from the primary nuclear transcript (Marillonnet et al. 2005). This involves the infiltration of plants via bacteria harboring a modified viral vector that contains silent nucleotide substitutions within viral RNA-dependent RNA polymerase (RdRp) coding sequence and multiple introns (Marillonnet et al. 2005). Magnifection equips the advantage of gene amplification, high expression yield, and post-translational capabilities that is suitable for the large-scale production of the target protein.

Given the pivotal role of the SFTSV envelope glycoprotein (Gn subunit) as a major antigenic component in virus entry and fusion, our study aimed to produce soluble Gn using a plant-based expression system. Subsequently, this protein was utilized as an antigen to establish a Gn-specific ELISA, serving as a diagnostic tool for assessing SFTSV seroprevalence.

## Materials and methods

### Generation of plasmids expressing recombinant SFTSV rGn

**Fig. 1:**
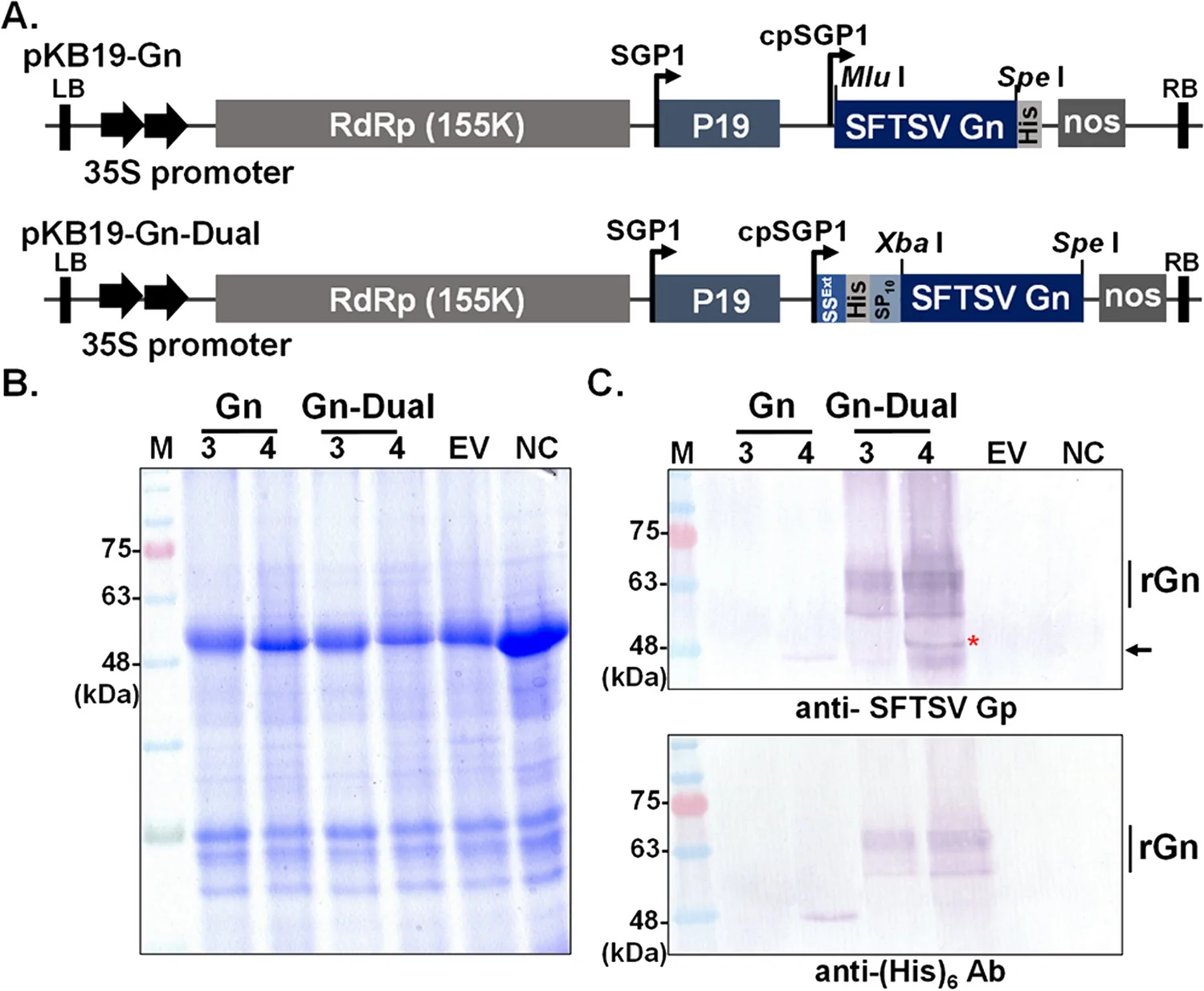
Expression of SFTSV Gn protein in *N. benthamiana* plants. **A** Schematic illustration of BaMV-based expression cassettes. Two constructs, namely pKB19-Gn and pKB19-Gn-Dual, which were derived from pKB19 vector, were generated for Gn expression. In the plasmid pKB19-Gn, the SFTSV Gn coding sequence was cloned into vector pKB19, containing BaMV RNA-dependent RNA polymerase gene (RdRp, 155 K), silencing suppressor P19 gene, and His-tag sequence. Expression of RdRp was under the control of a dual 35S promoter of Cauliflower mosaic virus (CaMV) and a nopaline synthase (nos) terminator of *Agrobacterium tumefaciens*. Moreover, to enhance the yield and secretory level of Gn protein, another construct named pKB19-Gn-Dual, which includes two additional elements, i.e., the signal peptide (SS.^Ext^) and 10 tandem repeats of Ser-Pro, (SP)_10_, at the N-terminal of Gn gene was generated. Expression of P19 and Gn was driven by the BaMV subgenomic promoter 1 (SGP1) and coat protein subgenomic promoter (cpSGP) as indicated by arrows. LB and RB indicate the left and right borders for the expression cassette, respectively. **B**-**C** Analysis of Gn expression in inoculated leaves. Total protein was prepared from *N. benthamiana* leaves, infiltrated with *A. tumefaciens* harboring either pKB19-Gn (Gn), or pKB19-Gn-Dual (Gn-Dual) at 3- and 4-day post inoculation (dpi) (lane 3, 4) and resolved by SDS-PAGE **B**, followed by western blot analysis using the antibody against Gp protein or His-tag **C**. Of note, plants infiltrated with either pKB19 (lane EV) or non-inoculate intact leaves (NC) serve as the negative control. The molecular weight of rGn monomer was predicted to be approximately 48 kDa (as indicated by an asterisk, or arrow)

Two BaMV-based expression vectors were used for the expression of rGn in plant system. Vector pKB19 contains elements, including BaMV RNA-dependent RNA polymerase (RdRp, 155 K), silencing suppressor P19 (519 bp, GenBank accession no. AJ288926) of *Tomato bushy stunt virus*, and the His-tag, as well as two copies of 35S promoters of Cauliflower mosaic virus (CaMV) and a nopaline synthase (nos) terminator of *Agrobacterium tumefaciens*, which controls the transcription of RdRp that in turns drives expression of subgenomic RNA (Chen et al. 2017). Moreover, to enhance the yield and soluble level of Gn protein, two additional elements, i.e., the signal peptide (SS^Ext^) and 10 tandem repeats of Ser-Pro, (SP)_10_ from hydroxyproline-O-glycosylated peptides (HypGPs), were included in another vector pKB19-Dual (Fig. 1A) (Jiang et al. 2020). The Gn coding region was inserted downstream of P19 in pKB19 vector or fused with the dual signal peptide sequences in pKB19-Dual vector. Of note, the transcription of P19 or rGn was under the control by the BaMV subgenomic promoter 1 (SGP1) and coat protein subgenomic promoter (cpSGP) are indicated by arrows (Fig. 1A).

Initially, the sequence (encoding residues 19 to 452, excluding the original signal peptide and transmembrane regions) of SFTSV Gn (strain TNB1580, GenBank Accession No. LC579716) was amplified by polymerase chain reaction (PCR) from the plasmid containing the SFTSV M segment, kindly provided by Dr. Ken Maeda. Two sets of Gn- specific primers, F-MluI-SFTSV-Gn (5′-GCACGCGTACCATgGGGGACACAGGCCCAATC)/R-SpeI-SFTSV-Gn (5′-GCACTAGTCTTCTTCGCAGGGTAGC), and F-XbaI-SFTSV-Gn-Dual (5′- GCTCTAGAGAGAACCTTTACTTTCAGGGAGGGGACACAGGCCCAATC)/ R-SpeI-SFTSV-Gn-Dual (5′-GCACTAGTTCACTTCTTCGCAGGGTAGC) were used to generate Gn subunit that were further trimmed by *Mlu* I/*Spe* I and *Xba* I/*Spe* I enzymes and inserted into the vectors pKB19 and pKB19-Dual, respectively. Sequences of the resulting plasmids pKB19-Gn and pKB19-Gn-Dual were confirmed by automated sequencing.

### Transient expression of rGn in *N. benthamiana* plants

Transient expression in *N. benthamiana* was performed via *Agrobacterium*-mediated infiltration following the protocol described in one previous report (Jiang et al. 2020). Briefly, plasmids were introduced to *Agrobacterium tumefaciens* (PGV3850) competent cells via electroporation. The bacteria harboring expression plasmid were resuspended in an agroinfiltration buffer (10 mM MgCl_2_ and 10 mM MES) to attain an optical density at 600 nm (OD_600_) of 0.5. Subsequently, the bacterial culture was infiltrated into 6-week-old *N. benthamiana* plants at the 5–6 leaf stage using a 1 mL syringe without a needle. Four infiltrated plants (Gn, Gn-dual, pKB-19, NC) were maintained in the greenhouse at a temperature of 28 °C for 2 to 5 days post-infiltration (dpi).

### Electrophoresis and western blot analysis

The protein expression profile was analyzed by sodium dodecyl sulfate–polyacrylamide gel electrophoresis (SDS-PAGE) and western blot analysesfollowing the procedure established in our laboratory (Liao et al. 2021). Specifically, total proteins were extracted from agroinfiltrated leaves at the time indicated in each experiment using the protein extraction buffer (50 mM Tris–HCl, pH 8.0, 10 mM KCl, 10 mM MgCl_2_, 1 mM EDTA, 20% glycerol, 2% SDS and 10% β-mercaptoethanol) at a ratio of 1:2.5 (w/v). The expression level and purity of rGn were revealed by SDS-PAGE and confirmed by western blot analyses using anti-SFTSV Gp (Novus Biological) and anti-His-tag as the primary antibodies.

### Large-scale production of rGn by agroinfiltration procedure

Time course analysis was conducted to evaluate the influence of agroinfiltration on rGn expression in *N. benthamiana* containing pKB19-Gn-Dual plasmid. *Agrobacterium* culture (250 mL) grown overnight at 28 °C (OD_600_ approximately 1.5 to 1.8) was harvested, and the resulting pellet was resuspended in the same volume of agroinfiltration buffer. To optimize the infiltration efficiency, the *Agrobacterium* harboring the pKB19-Gn-Dual vector was further diluted with agroinfiltration buffer to achieve an OD_600_ ranging from 5.00E-01 to 5.00E-04. To inoculate the entire plant, 8-week-old *N. benthamiana* plants cultivated in the greenhouse were utilized. Infiltration of individual leaf sectors was performed by using a vacuum-infiltration. Alternatively, for whole plant inoculation, vacuum infiltration was carried out following previous protocols and modifications developed by our research team (Jiang et al. 2020; Leuzinger et al. 2013). In brief, a beaker containing the inoculation solution was placed in a vacuum chamber (30 × 20x20 cm) with the aerial parts of a plant immersed in the solution. A vacuum of 25 to 30 mmHg was applied for 1 min. Upon release of the vacuum, the inoculation solution rapidly infiltrated all submerged *N. benthamiana* in whole plant leaves. Subsequently, the ninety-six infiltrated plants were maintained in a growth chamber at 28 °C, with 16 h of light and 8 h of darkness. To monitor the protein expression status, initially, a total of 0.1 g of infiltrated leaves were harvested at various days post-infiltration (dpi), and subsequently, the protein level was analyzed by western blotting with antibodies against SFTSV Gp before the final harvest for protein purification.

### Extraction of rGn

For large-scale expression, *N. benthamiana* leaves were inoculated with *Agrobacterium* harbor pKB19-Gn-Dual expressed plasmid and were collected at 5 dpi. The infected leaves were frozen at -80 °C for overnight. The frozen 100 g of leaves were manually crushed before adding 200 ml of buffer A [50 mM Tris–HCl, pH 8.0, 120 mM KCl, 15 mM MgCl_2_, 20% glycerol, 0.1% β-mercaptoethanol, 0.1% Tween 20, 0.1% PMSF, and 1 tablet of protease inhibitor (Roche Life Science, Penzberg, Germany)]. The leaf homogenate was then filtered through a layer of Miracloth (Calbiochem, La Jolla, CA), and centrifuged 4 °C for 10 min at 1000 × *g* to obtain the pellet (P1) and the supernatant (S1) fractions. The P1 fraction contained the cell wall, nuclei, and chloroplasts from the homogenized cells. The S1 fraction was subjected to further centrifugation at 30,000 × *g*, 4 °C for 30 min to separate the soluble cytosolic (S30) and the precipitated membrane fraction (P30). Next, the abundant ribulose-1,5-bisphosphate carboxylase/oxygenase (RuBisCO) protein was removed following the isoelectric precipitation; to do so, the pH of the S30 fraction was adjusted to 5.1 by acetic acid (AcOH). Samples were then centrifuged at 30,000 × *g* for 30 min to separate the pellet (Pa fraction containing RuBisCO) and supernatant fractions (Sa), the Sa fraction was adjusted to pH 7.4 by adding 10 N NaOH while keeping on ice for further purification. Protein contents in all fractions were subjected to electrophoresis and western blot analysis.

### The purification of rGn by a two-step chromatographic process utilizing IMAC and HIC

The rGn protein was purified by immobilized metal affinity chromatography (IMAC) and hydrophobic interaction chromatography (HIC). Initially, IMAC was performed on NuviaTM IMAC Ni-Charged resin (Bio-Rad) according to the manufacturer’s instructions. Briefly, the supernatant fractions extracted after acid treatment (Sa) were clarified by a 0.45 μm filter (Pall Corporation) and then applied to the column followed by a binding step using a peristaltic pump at a flow rate of 1 mL/min for overnight at 4 °C. The column was washed with 10 column volumes of wash buffer (50 mM Tris–HCl, pH 8.0, 120 mM KCl, 15 mM MgCl_2_, 20% glycerol, 20 mM imidazole), and proteins were sequentially eluted with elution buffer (50 mM Tris–HCl, pH 8.0, 120 mM KCl, 15 mM MgCl_2_, 20% glycerol, 300 mM imidazole) into 25 fractions (1 mL per fraction). All fractions from IMAC were analyzed by western blot analysis, and the high-yield fraction was harvested for the following HIC process.

Purification by HIC was carried out in a HiTrap Phenyl HP column (Merck) through an AKTA Purifier GE Healthcare system, following the manufacturer’s manual. The entire process, including samples and buffer, was filtered using a 0.22-μm filter. Briefly, the column was equilibrated with buffer A containing 1 M ammonium sulfate. The high-yield fraction was then supplemented with ammonium sulfate to a final concentration of 1 M and injected into the column, followed by a cycle binding incubation for 1 h at 4 °C. After the column was washed with 3 column volumes of 1 M ammonium sulfate of phosphate-buffered saline (PBS), the target protein was eluted using PBS buffer containing a linear gradient of 1 M to 0 M ammonium sulfate. All eluted fractions were first equilibrated with ammonium sulfate for the final elution process by PBS and analyzed by western blot. The identity and glycan profile were determined by LC–MS/MS by Glycan Sequencing/Profiling Core Facility, Genomics Research Center, Academic Sinica, Taiwan.

### Estimation of the protein yield

We initially determined rGn protein concentration by quantifying signal intensity relative to known BSA concentrations on the gel. In brief, a standard curve was established using BSA standards with known concentrations, and the protein signal was converted to pixel values by ImageJ software. The protein concentration was then estimated by referencing pixel values to the BSA standard curve. Ultimately, the approximate yield of protein can be estimated in considering the total volume of protein purified from 100 g of leaves.

### Expression of SFTSV Gp in mammalian cells

Construct expressing SFTSV Gn protein (residues 19 to 452, excluding the original signal peptide and transmembrane regions) was transiently transfected into human embryonic kidney (HEK) 293 T cells by lipofectamine 2000 (Invitrogen) following manufacturer’s instructions (Chung et al. 2022). In brief, 2 μg of plasmid DNA diluted with serum-free DMEM was mixed with DMEM-diluted 2 μL of lipofectamine 2000. Subsequently, the DNA-liposome mixture was added into HEK 293 T cells seeded in a 12-well plate. The cells were harvested at 48 h after transfection followed by protein analysis.

### Field animal samples

A total of sixty-four monkey serum samples were collected in Ube-shi, Yamaguchi Prefecture, Japan. All the serum samples of wild monkeys were provided by hunters who caught wild monkeys for nuisance control under the Program of Prevention from the Bird and Animal Damages from November 2013 to February 2016 (license number: Shimonoseki-No.24 and 26). The SFTSV infection status of these samples was initially determined by SFTSV-infected cell lysate-based ELISA (ICL-ELISA) (Tatemoto et al. 2022).

### Establishment of rGn ELISA

The rGn-ELISA was established using rGn expressed in plants. Antigen rGn (or SFTSV HB29-infected vs. mock-infected HuH-7 cells in case of ICL-ELISA) at concentrations of 50 ng, 100 ng, and 200 ng were coated using coating buffer (0.05 M carbonate-bicarbonate buffer, pH 9.6) in an ELISA plate (Thermo Scientific™). After incubation at 4 °C for overnight and blocking using 1% Block Ace (BA) (KAC) in PBS at 37 °C for 30 min, 100 µl of the serum in 1:100 dilution in 0.4% BA in PBST (PBS containing 0.05% tween 20) was added into each well and incubated at 37 °C for 30 min, followed by three washes with PBST. Subsequently, the secondary antibody, which was Protein A/G conjugated with HRP (Invitrogen) diluted in 0.4% BA was added and reacted for at 37 °C for 30 min. After three washes with PBST, the signals were visualized by ABTS Peroxidase Substrate (SeraCare Life Science). The signal was measured by a microplate reader (Bio-Rad, Hercules, CA, USA) using a 405-nm filter. A commercial anti-SFTSV Gn antibody (Gp41153, Novus Biological) and mouse polyserum specific for rGn (manufactured by LTK bioLaboratories, Taiyuan, Taiwan) was included as positive control antibody to optimize the detection conditions.

### Receiver-operating characteristic (ROC) curves analysis and statistical analysis

The performance of rGn ELISA was evaluated by receiver-operating characteristic (ROC) curves. The area under the curve (AUC) and the kappa coefficient (κ) were used to determine the consistency between ICL-ELISA (the reference method) and rGn-ELISA. Statistical analyses were conducted by using SPSS (version 20) and SAS statistical software (version 9.4). A *p*-value less than 0.05 was defined as statistically significant.

## Result

### Construction of chimeric BaMV cassettes and the expression of rGn

To overexpress the recombinant SFTSV Gn (rGn) in soluble form, the BaMV-based vector system was exploited (Jiang et al. 2020, 2019). Initially, SFTSV Gn coding sequences were inserted into BaMV expression cassette, and the resultant plasmid was designated as pKB19-Gn (Fig. 1A). Furthermore, the secretory signal (SS^Ext^) of *N. benthamiana* extensin protein and the sequences of 10 tandem repeats of the “Ser-Pro” motif, (SP)_10_, (Jiang et al. 2020; Xu et al. 2007) were adopted herein to improve the expression of soluble form of rGn (shown as pKB19-Gn-Dual in Fig. 1A). Both expression cassettes were individually infiltrated into *N. benthamiana* leaves using *Agrobacterium*-mediated inoculation (agroinfiltration). The relative expression level of SFTSV Gn driven by the two constructs were analyzed (Fig. 1B–C).

Despite the absence of apparent rGn overexpression (Fig. 1B), the immunoblotting results clearly indicated that incorporation of dual elements, i.e., SS^Ext^ and (SP)_10_ indeed significantly elevated rGn expression (Fig. 1C). Notably, the pKB19-Gn vector exhibited a very low level of rGn protein expression, which was detectable at 4 dpi (Fig. 1C, lane 4 in the pKB19-Gn construct). In contrast, the fusion of the two signal tags enabled the ready detection of high levels of rGn as early as 3 dpi. Of note, due to the inclusion of several fusion tags, the size of rGn protein expressed from pKB19-Gn-Dual construct is slightly larger than that from pKB19-Gn. Ultimately, pKB19-Gn-Dual construct was selected for large-scale production of rGn in *N. benthamiana*. Moreover, as indicated in a time-course experiment, higher doses or extended-expression led to leaf discoloration and necrosis (Supplementary Figure S1A), and the highest expression level of rGn was observed when leaves were infiltrated with 5E-02 of the culture and harvested at 5 dpi (supplementary Figure S1B).

### Glycosylation of rGn in *N. benthamiana*

**Fig. 2:**
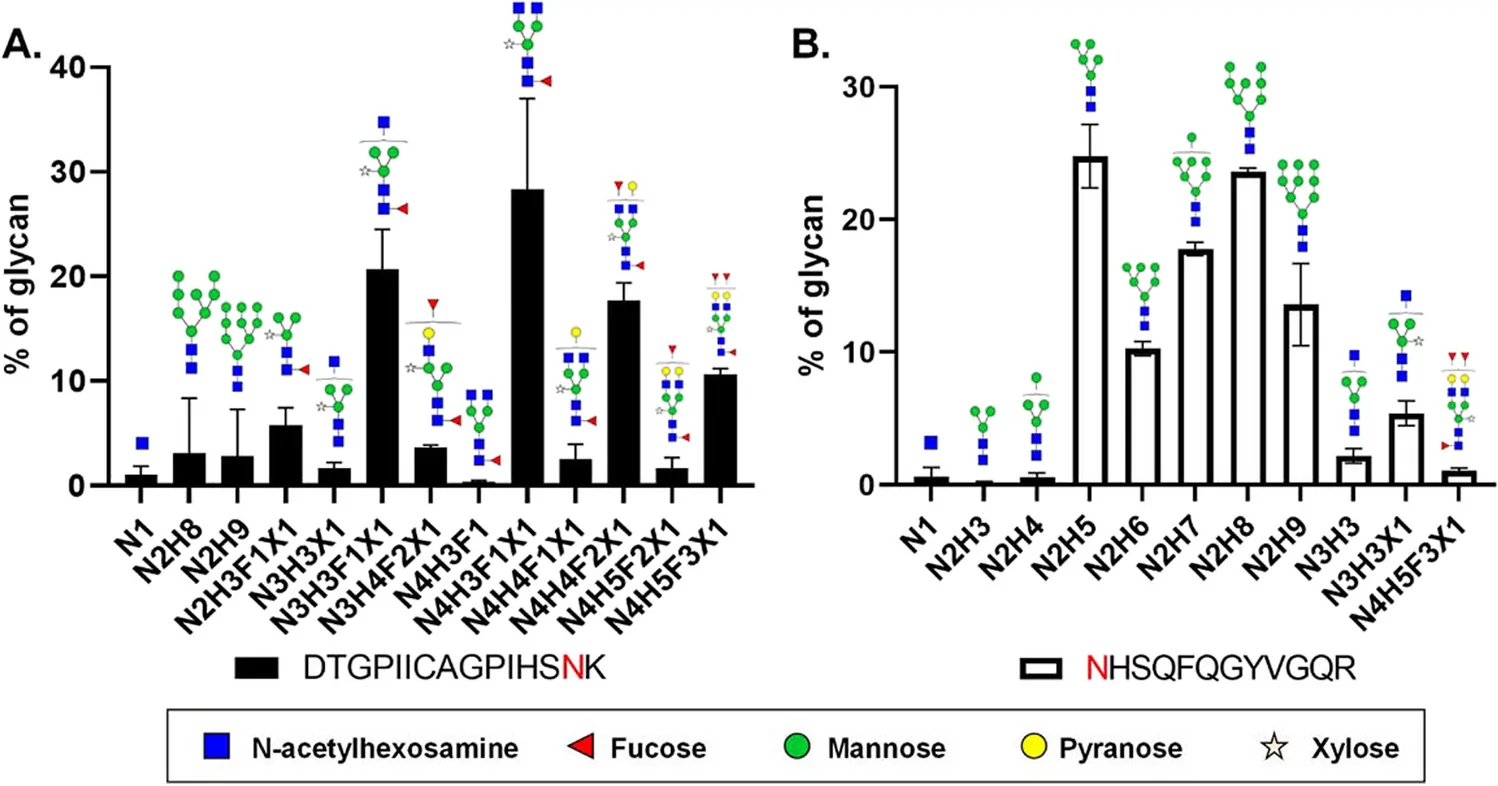
Analysis of the glycan profile of the rGn protein. The glycosylation profile of rGn protein was verified by LC–MS/MS. Two tryptic-digested glycopeptides, namely, DTGPIICAGPIHSNK (residues 2–16, in panel A) and NHSQFQGYVGQR (residues 45–56, in panel B), originating from the SFTSV Gn protein, were successfully identified for glycosylation composition analysis. Three batches of rGn were analyzed and the average percentage and the symbol of various glycan types are shown. The abbreviations indicated in X-axis for monosaccharides are H, hexose; N, N-acetylhexosamine; F, fucose; X, xylose

It is important to highlight that aside from the Gn monomer, approximately 48 kDa (as indicated by an asterisk), a multitude of signals with molecular weights ranging from 48 to 70 kDa were also observed at 3 or 4 dpi (Fig. 1C, lanes 3 and 4 in pKB19-Gn-Dual construct). As predicted using the NetNGlyc 1.0 program (http://www.cbs.dtu.dk/services/NetNGlyc/), two N-glycosylation sites (N^15^KS, N^45^HS) are present in the Gn protein (Supplementary Figure S2). It is likely the multiple bands were glycosylated forms of rGn. Therefore, the amino acid sequence and glycosylation state of plant-derived rGn was further verified by liquid chromatography-tandem mass spectrometry (LC–MS/MS) analysis. Overall, coverage of rGn was 84.79% (Supplementary Figure S2C), and two tryptic-digested glycopeptides, specifically DTGPIICAGPIHSN^15^K (residues 2–16, Fig. 2A) and N^45^HSQFQGYVGQR (residues 45–56, Fig. 2B), originating from the SFTSV Gn coding region, were successfully identified. The most abundant glycan detected at the N^15^ site was of the fucosylated complex-type structure, including N4H3F1X1 and N3H3F1X1, whereas the major glycan attached to the N^45^ site was the non-fucosylated complex-type, such as N2H5, and N2H8. The major proportion of N glycan occupied by complex-type glycan structure indicates that the rGn protein was fully glycosylated during the intracellular transportation through the secretory pathway.

### Purification of rGn by acid precipitation coupled with two-step chromatography

Next, the production and purification of rGn protein were carried out on a large scale. As indicated in a time-course experiment, the highest expression level of rGn was observed when leaves were infiltrated with 5E-02 of the culture and harvested at 5 dpi (supplementary Figure S1). Subsequently, the purification of SFTSV rGn protein was conducted through a series of meticulous procedures, following the strategies optimized in our team (Jiang et al. 2020).

**Fig. 3:**
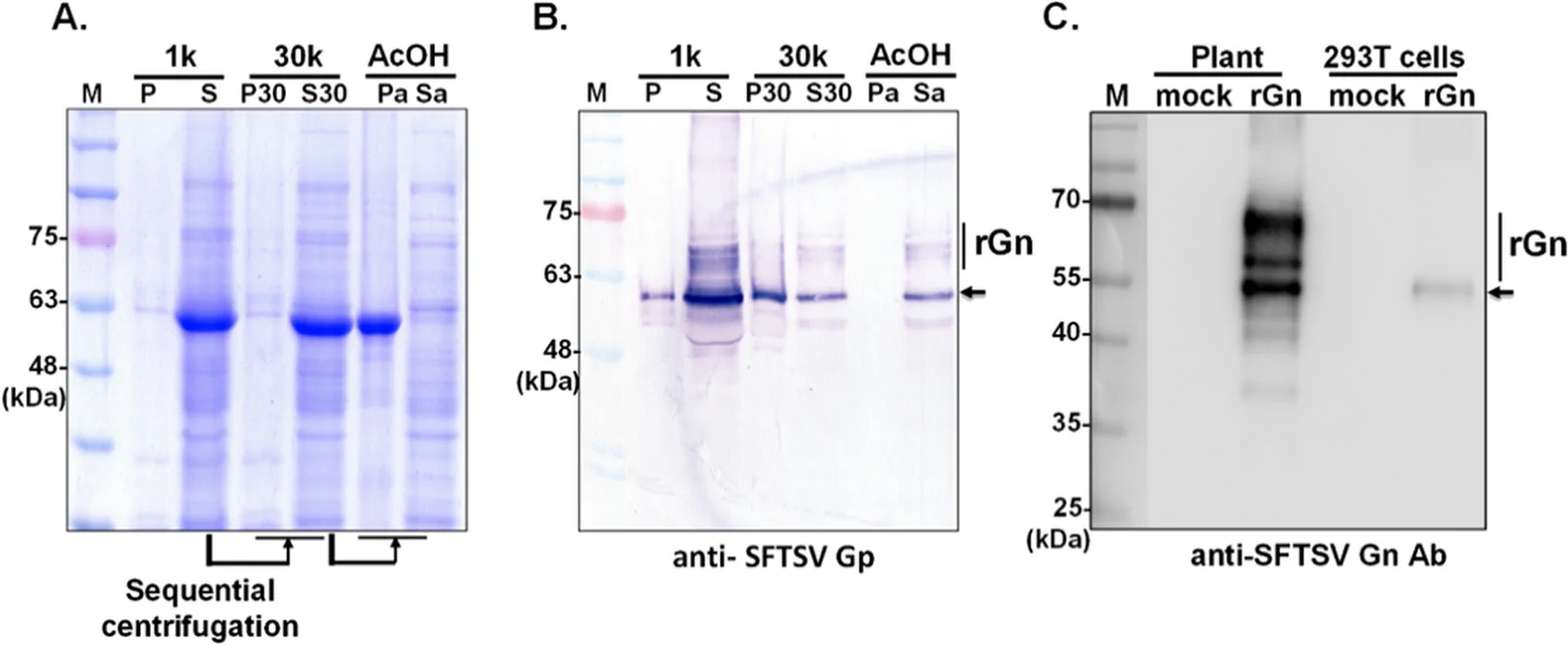
Preparation of crude extract containing soluble SFTSV rGn protein from *N. benthamiana* leaves. Homogenates of leaves agroinfiltrated with pKB19-Gn-Dual construct were initially centrifuged at 1,000 × *g* (1 k) to separate the pellet (P1) and supernatant (S1) fractions. Subsequently, the S1 fraction underwent further centrifugation at 30,000 × *g* (30 k) to fractionate the membranous-bound (P30) and soluble protein (S30) components. The soluble proteins (S30) were then subjected to acetic acid (AcOH) treatment to remove RuBisCO (Pa fraction), resulting in the soluble fraction (Sa). The figure illustrates the overall protein profile **A** and the presence of rGn, as detected by immunoblot assay **B**. Expression of rGn in plant system was compared with that produced in human 293 T cells **C**. Monomer rGn was indicated as arrowheads

Initially, *N. benthamiana* leaf homogenates were pre-clarified by sequential centrifugations at 1000 × *g* (1 K) followed by 30,000 × *g* (30 K) to obtain soluble protein fractions (lane S30, in Fig. 3A). Noticeably, the large subunit of RuBisCO, with a molecular weight of 53 kDa, was prominently present as the endogenous protein in the crude extract from vacuum-infiltrated leaves (Fig. 3A). To mitigate the possible interference of the non-targeted protein (specifically RuBisCO) in the course of rGn protein purification, acetic acid treatment was conducted. This method has been demonstrated to effectively precipitate RuBisCO (Park et al. 2015), thereby facilitating its removal from the soluble fraction. Following acetic acid treatment, the content of RuBisCO protein in the soluble fraction (indicated as lane Sa, in Fig. 3A) was remarkably reduced when compared with the original S30 fraction. It is worth highlighting that the majority of RuBisCO was retained in the precipitate (lane Pa, in Fig. 3A). Immunoassay confirmed the presence of soluble rGn within the Sa fraction (lane Sa, in Fig. 3B).

**Fig. 4:**
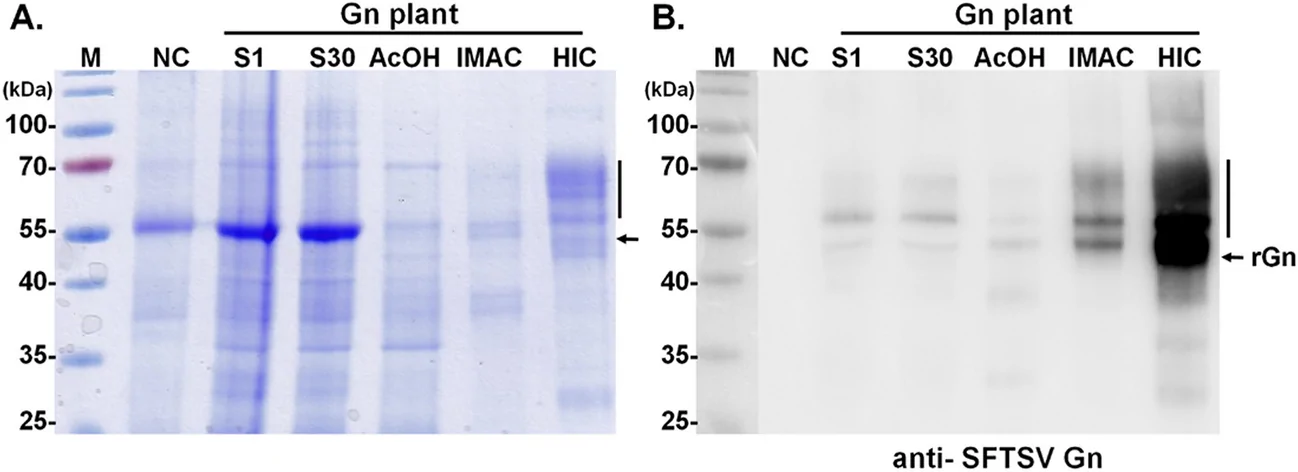
Purification of the rGn produced from *N. benthamiana* leaves. The soluble protein (S) was initially fractionated from 100 g of *N. benthamiana* infiltrated leaf tissue at 5 dpi. Subsequently, the soluble rGn underwent acetic acid (AcOH) precipitation, followed by purification using IMAC and HIC techniques. The quality and integrity of the purified pGn were evaluated through SDS-PAGE **A** and confirmed by Western blot analysis using SFTSV Gn-specific antibodies **B**

Subsequently, soluble fraction containing rGn underwent additional purification via a two-step chromatographic process. This involved an initial IMAC step, which capitalizes on the presence of the six-histidine tag at N-terminus of rGn protein, followed by HIC that facilitates the depletion of polysaccharides compounds, such as pectins and arabinogalactan glycoproteins, from leaf homogenate. Following these two chromatographic procedures, the yield of rGn was substantially enriched by IMAC and HIC steps (Fig. 4); and the estimated overall yield was approximately 7 mg/kg from infiltrated *N. benthamiana* leaves (Supplementary Figure S3). The authenticity of rGn proteins, with anticipated sizes as purified via HIC (as illustrated in Fig. 4A), was subsequently validated through immunoblotting using antibodies against SFTSV Gn (Fig. 4B).

### Application of rGn expressed in plant to establish ELISA (rGn-ELISA) for the detection of SFTSV antibody

Next, given the notably lower yield of soluble recombinant Gn protein expressed in mammalian cells compared to the production in plants (Fig. 3C), we endeavored to establish an enzyme-linked immunosorbent assay (ELISA) employing rGn protein purified from infiltrated *N. benthamiana* leaves. This ELISA was designed to monitor the seroprevalence of SFTSV, an emerging infectious disease in Taiwan.

Initially, ELISA optimization involved testing various concentrations (50, 100, and 200 ng/well) of the coating antigen (rGn) against a range of diluted positive antibodies specifically reactive to the SFTSV Gn protein. These antibodies included the polyserum derived from mice immunized with Gn protein produced in mammalian cells, and a commercially available antibody designed for the SFTSV G protein. In the indirect ELISA utilizing rGn protein, both positive control antibodies were successfully detected rGn protein in a dose dependent manner.

**Fig. 5:**
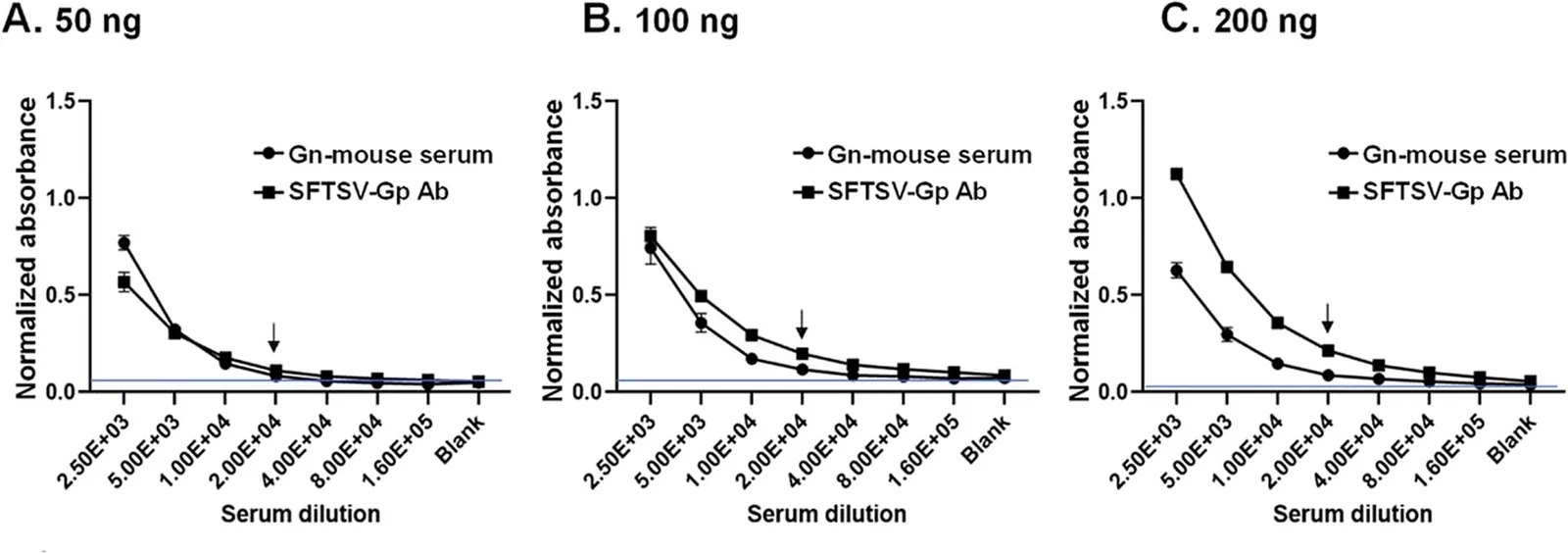
Establishment of enzyme-linked immunosorbent assay (ELISA) system for Gn antibody detection using Gn protein expressed in the plant. In the pursuit of optimizing the Gn-ELISA, two Gn-specific antibodies including the Gn-immunized mouse serum (denoted as Gn-mouse serum) and a commercial SFSTV Gp antibody, serving as positive control antibody, were used to optimize the Gn-ELISA. The Gn protein, purified from infiltrated plants, was coated onto the plate at concentrations of 50, 100, and 200 ng per well. The well without protein coating served as a blank control. Absorbance values were subsequently normalized with the negative control. The optimal condition was identified as the reaction of the serum at a 1:5000 dilution of the positive antibody from mice immunized with rGn (produced by LTK bioLaboratories, Taiyuan, Taiwan), along with the commercial polyclonal antibody, utilizing 200 ng of the antigen. The data from four independent replicates are depicted in the plot, with corresponding standard deviation (SD) bars. The cut-off value was established by setting the mean OD of blank samples plus 5 SD as denoted by the blue line. The last dilution of antibodies that showed a significant difference from the blank control was indicted as arrow signs

As illustrated in Fig. 5, the antibody response against rGn consistently diminished across all serum samples with each subsequent dilution. However, the positive signal remained detectable when Gn-mouse polyclonal serum and a commercial SFTSV-Gp antibody were diluted from 1:2500 to 1:16000. Given the potential for unexpected SFTSV infection in field animal samples, the subsequent validation experiments employed a higher concentration of 200 ng of rGn per well to ensure the sensitivity.

### Validation of rGn-ELISA by comparison with standardized ELISA

**Fig. 6:**
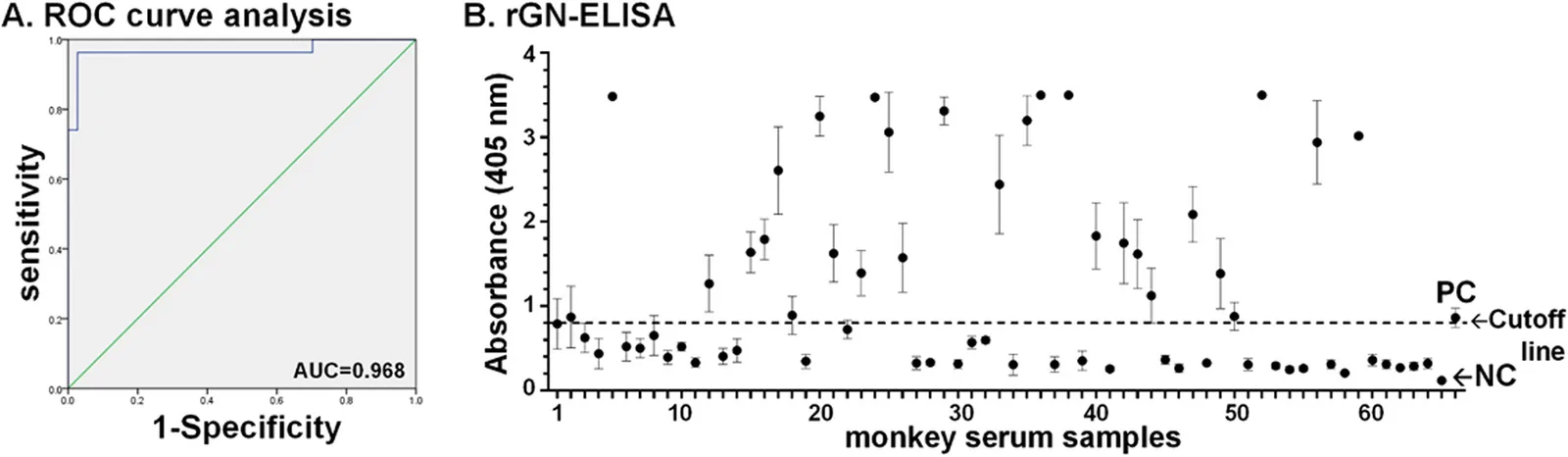
Validation of in-house rGn ELISA by detection of field monkey serum samples. **A** Initially, the diagnostic performance of rGn-ELISA on antibody detection was validated. Receiver operating characteristic (ROC) curve analysis was performed to evaluate the diagnostic accuracy of the newly developed Gn-ELISA (with 200 ng of the coating antigen) in comparison to the established standard ELISA, known as ICL-ELISA, employing SFTSV-infected cell lysate as the coating antigen. This analysis encompassed a comparison of the areas under the curve (AUC) for the rGn-ELISA in relation to the ICL-ELISA. The 95% confidence interval (95% CI) was ascertained to be 0.916–1.0, signifying a high level of confidence in the diagnostic performance assessment. **B** Anti-SFTSV Gn antibodies were detected in sixty-four monkey serum samples through the rGn-ELISA method based on the cutoff value, represented by the dashed line at 0.8, which was determined through ROC curve analysis. The data from three independent replicates are depicted in the plot, with corresponding standard deviation (SD) bars

Subsequently, rGn-ELISA was employed to detect SFTSV Gn antibodies in serum samples derived from field monkeys. To validate the results obtained from in-house rGn-ELISA, the SFTSV antibody status in all field monkey sera was initially confirmed using a standard ELISA known as ICL-ELISA, which utilized whole infected cell lysate as the coating antigen (Kimura et al. 2018). Initially, using the ICL-ELISA as the gold standard, we conducted receiver operating characteristic (ROC) curve analysis to assess the discriminative power of the rGn-ELISA. As demonstrated in Fig. 6A, the area under the curve (AUC) values, which reached 0.968 (with a 95% confidence interval of 0.916–1.0), demonstrate a high degree of concordance between the results obtained from the rGn-ELISA and those derived from the standard method.

Subsequently, a cut-off value of 0.87 was determined based on ROC curve analysis (Fig. 6A). Notably, this cut-off value led to significant discrimination between the positive and negative groups, with a *p*-value < 0.05. This strong statistical significance highlights a robust correlation between the two testing methods. Among the sixty-four serum samples analyzed via rGn-ELISA (as shown in Fig. 6B), twenty-eight samples (accounting for 44%) tested positive, while thirty-six sera (56%) tested negative. However, three samples displayed discrepant results as validated with ICL-ELISA: two were false negatives, and one was a false positive. Further evaluation of specificity and sensitivity, conducted through Pearson’s chi-squared test for rGn-ELISA and ICL-ELISA, revealed values of 94% and 96%, respectively. Moreover, the Kappa coefficient, with a value of 0.904 (indicating excellent agreement when > 0.75; perfect agreement = 1) demonstrated a high level of consistency between these two methods.

## Discussion

Since its initial discovery in Taiwan in 2019 (Lin et al. 2020), nationwide surveillance of SFTSV is imperative (Kuan et al. 2023). Leveraging the sophisticated eukaryotic expression system, this study has successfully achieved the bulk purification of the recombinant SFTSV rGn, produced in *N. benthamiana* leaves through agroinfiltration, driven by BaMV viral protein expression systems. Importantly, rGn is utilized to develop a user-friendly ELISA system for assessing SFTSV seroprevalence.

Seroprevalence requires manipulation of viral particles or infected cell lysate serving as antigen (Kimura et al. 2018). To minimize the risk derived from handling SFTSV, a Risk Group 3 (RG-3) pathogen, and to produce Gn proteins faithfully represent the original antigenicity, plants equipped with essential post-translational modifications (PTM) (Benchabane et al. 2009; Xu et al. 2007), was selected. SFTSV Gn is a glycoprotein, making translation machinery with PTM the most suitable choice. Consequently, efforts were made to express the Gn protein in both mammalian cells and plants. Notably, the yield of rGn in plants surpassed that in the mammalian system (Fig. 3C), and as illustrated in Fig. 3C, the transient expression of rGn protein in leaves using BaMV viral vectors yielded substantial material, with a total yield of 7 mg/kg of leaves (Supplementary Fig. 3). Moreover, it is worth noting that plant maintenance does not involve costly reagents, for instance, bovine serum, and *N. benthamiana* is a fast-growing option, as compared with mammalian cells. Therefore, the expression in tobacco leaves proves to be cost-effective and less time-consuming. These factors contribute to the scalability of this system, ensuring the production of abundant antigen levels and making it well-suited for the bulk production of glycoproteins.

Several strategies were developed to elevate the level or quality of recombinant protein expressed in plant systems. As known secretory signals play a pivotal role in guiding proteins along the secretory pathway to the extracellular space (Anelli and Sitia 2008). To enhance the yield of soluble protein and streamline the subsequent purification process, rGn was expressed as a fusion protein with dual signal tags: the SS^Ext^ peptide and the (SP)_10_ motif at its N-terminus that have been proved to improve the efficiency of human IFN-γ secretion (Jiang et al. 2020). Furthermore, the impact of the (SP)_10_ dipeptide repeat has been shown. For instance, when enhanced green fluorescence protein (EGFP) was expressed as a fusion with (SP)_32_, consisting of 32 repeats of the SP dipeptide, a substantial increase in yields was observed (Dolan et al. 2014). Also, the inclusion of 20 tandem repeats of SP dipeptide remarkably increased the total expression level and the secretory yield without affecting the function of the stem cell factor (Wang et al. 2021). Consistently, this current investigation demonstrated that the expression level of untagged rGn, driven by the pBK19 construct, remained below the detection threshold (Fig. 1C). However, by incorporating SS^Ext^ and (SP)_10_ sequences, a substantial enhancement in rGn protein accumulation was achieved, thereby alleviating the challenges associated with subsequent purification.

The molecular weight of the SFSTV Gn monomer was estimated to be approximately 48 kDa. However, a multitude of rGn signals ranging from 50 to 75 kDa were detected by the SFTSV Gp-specific antibody (Fig. 3C). Structural analysis has identified two N-linked glycans (N33 and N63) located in subdomain I of the SFTSV Gn protein (Wu et al. 2017). Consistently, the glycosylation of two asparagine residues (N15, N45) of plant-made rGn was confirmed by LC–MS/MS analysis (Fig. 2). Moreover, based on their glycan profile, the fucosylated complex-type structure was the most abundant glycan detected at the N15 site indicating that the rGn protein was fully glycosylated during the intracellular transportation through the secretory pathway. This result supports the plant is an ideal system for glycoprotein expression.

Among the 64 monkey sera analyzed, three samples exhibited discrepant outcomes between the two ELISA methods. Two of these samples were determined to be false positives by rGn-ELISA. It is worth noting that the normalized absorbance values of these two samples, i.e., 0.87 and 1.57, respectively, were just below the 0.87 cut-off value when tested by the reference method rGn-ELISA. This discrepancy is likely attributable to differences in coating antigen levels, with the rGn-ELISA utilizing a relatively higher concentration of purified rGn (200 ng) compared to the Gn protein content in infected cell lysates (data not shown), potentially leading to variations in sensitivity. On the other hand, ICL-ELISA incorporates antigens derived from a whole panel of viral proteins, including Gn and NP, the latter being another immunodominant protein that stimulates a significant antibody response (Umeki et al. 2020). Therefore, we hypothesize that the false negative result from the rGn-ELISA detection may be due to differences in the antigen composition between the two ELISA systems. Employing ELISA with multiple immunodominant viral antigens (for instance, Gn combined with NP) could enhance sensitivity, provided a sufficient level of antigens is applied in the system. Nevertheless, the high degree of concordance between the results obtained from the rGn-ELISA and those derived from the standard method, as indicated by the AUC values of 0.968 (with a 95% confidence interval of 0.916–1.0), is noteworthy. However, it is important to consider the potential variation in Gn protein sequences among different viral strains. Therefore, further evaluation of the cross-reactivity of rGn-ELISA with other SFTSV strains is warranted.

It is worth noting that currently available immunoassays for SFTSV antibody detection utilize antigens derived from either infected cell lysate or virus particles. While these antigens maintain the integrity of antigenicity, they require P3-containment facilities for handling such a highly pathogenic etiologic agent. In contrast, our novel ELISA system utilizes rGn protein, expressed in plants, as the coating antigen. This innovative approach successfully detected SFTSV antibodies in serum samples collected from naturally infected monkeys. As validated against a reference method that utilizes infected cell lysate (ICL-ELISA), the rGn-ELISA exhibited high specificity and sensitivity, with values of 94% and 96%, respectively.

In conclusion, we have effectively established ELISA systems for the precise detection of antibodies against SFTSV Gn, utilizing an innovative plant platform within the BaMV-expressed system. Our rGn-ELISA has been validated against a panel of field serum samples using a standard reference method. It exhibits high sensitivity and specificity, making it a valuable tool for detecting anti-SFTSV antibodies in a laboratory setting without the necessity for a Biosafety Level 3 (BSL-3) facility.

## Supplementary Information

Below is the link to the electronic supplementary material.

Supplementary file1 (PDF 893 KB)

## Data Availability

The data generated and/or analyzed during the current study are available from the corresponding author upon reasonable request.
