# Supplementary material for: Gn protein expressed in plants for diagnosis of severe fever with thrombocytopenia syndrome virus
Source: Appl Microbiol Biotechnol. 2024 Apr 19;108:303. doi: 10.1007/s00253-024-13135-0 (PMC11031438; doi:10.1007/s00253-024-13135-0)
Supplement: Supplementary file 1 [file 253_2024_13135_MOESM1_ESM.pdf]

**Supplementary figures**

**Gn protein expressed in plants for diagnosis of severe fever with thrombocytopenia syndrome virus**

**Yu-Chih Chang[1,2] · Hiroshi Shimoda[3] · Min-chao Jiang[1] · Yau-Heiu Hsu[4] · Ken Maeda[5]· Yumiko Yamada[2] · Wei-Li Hsu[1,2,6]***

[1] Doctoral Program in Microbial Genomics, National Chung Hsing University and Academia Sinica, Taichung, Taiwan

[2] Graduate Institute of Microbiology and Public Health, College of Veterinary Medicine, National Chung Hsing University, Taichung, Taiwan

[3] Laboratory of Veterinary Microbiology, Joint Faculty of Veterinary Medicine, Yamaguchi University, Yamaguchi, Japan

[4] Graduate Institute of Biotechnology, National Chung Hsing University, Taichung, Taiwan

[5] National Institute of Infectious Disease, Tokyo, Japan

[6] The iEGG and Animal Biotechnology Center, National Chung Hsing University, Taichung 402

*Corresponding author: Wei-Li Hsu, wlhsu@dragon.nchu.edu.tw

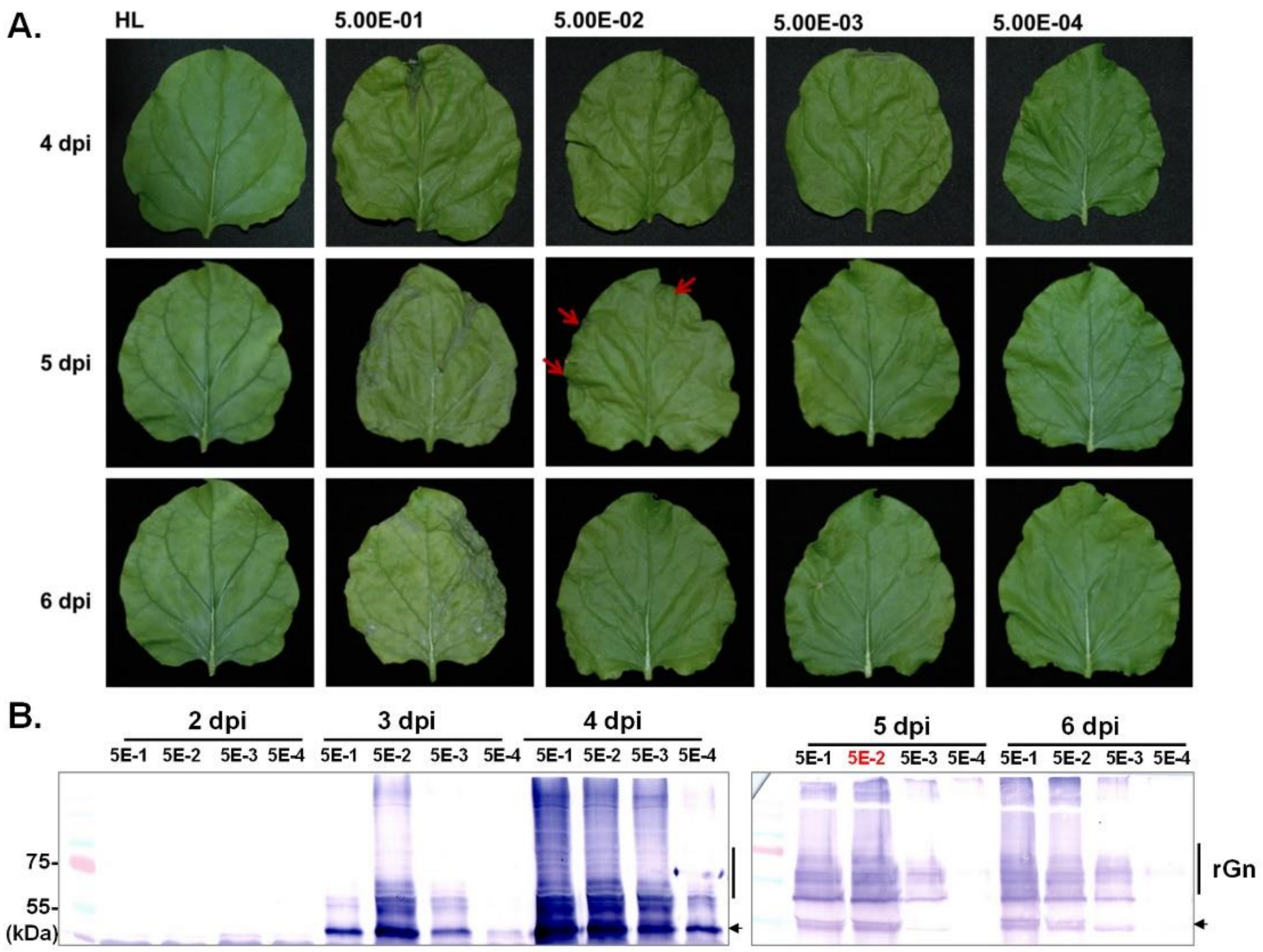


**Supplementary Figure S1. Optimization of agroinfiltration and expression condition for rGn. (A)** The phenotype of *N. benthamiana* leaves after 4-6 days post inoculation with *A. tumefaciens.* Leaves were inoculated with various dosages of *A. tumefacien*, ranging from OD of 0.5~0.0005 (labelled as 5.00E-01~5.00E-04) for 6 days. Severe symptoms appeared on fresh leaves inoculated with *A. tumefacien* at an OD of 5.00E-01at 4-6 days post-infection (dpi). In brief, leaves showed shriveling and necrotic symptoms at 4 dpi, and by 6 dpi, the lesion area increased along with yellowish/necrotic symptoms. An OD of 5.00E-02 caused viral symptoms with yellowish/light lesions (red arrow) at 2 dpi. HL, health leaves. (B) The expression level of rGn harvested from 2 dpi to 6 dpi was detected by western blot analysis using anti-Gn antibody. The rGn monomer and glycosylated rGn were indicated by arrows and lines, respectively. The highest expression level of rGn was observed when leaves were infiltrated with 5E-02 at 5 dpi (marked in red).

**A.** MGKMASLFATLLVVLVSLSLASESSA HHHHHH GG SPSPSPSPSPSPSPSPSPSP ENLYFQG
GDTGPIICAGPIHS**NKS**ADIPHLLGYSEKICQIDRLIHVSSWLR**NHS**QFQGYVGQRGGRSQV
SYYPAENSYSRWSGLLSPCDADWLGMLVVKKAKGSDMIVPGPSYKGKVFFERPTFDGYVG
WGCGSGKSRTESGELCSSDSGTSSGLLPSDRVLWIGDVACQPMTPIPEETFQELKSFSQSEF
PDICKIDGIVFNQCESESLPQPFDVAWMDVGHSHKIIMREHKTKWVQESSSKDFVCYKEGT
GPCSESEEKACKASGSCRGDMQFCKVAGCDHGEEASEAKCRCSLVHKPGEVVVSYGGMRV
RPKCYGFSRMMATLEVNPPERRIGQCTGCHLECINGGVRLITLTSELKSATVCASHFCGSATS
GKKNTEIQFHSGSLVGKTAIHVKGALVDGTEFTFEGSCMFPDGCDAVDCTFCREFLKNPQCY
PAKK*

**B.**

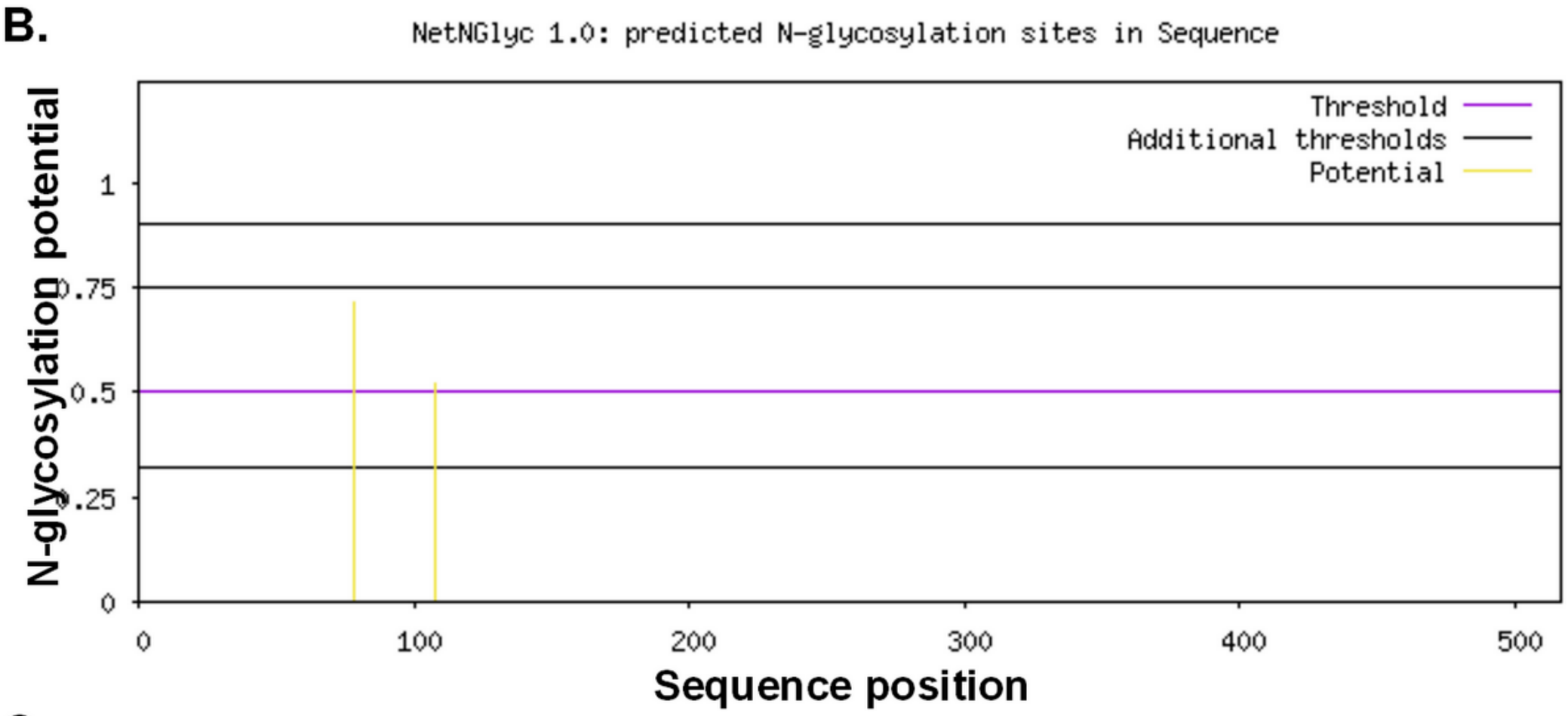


**C.**

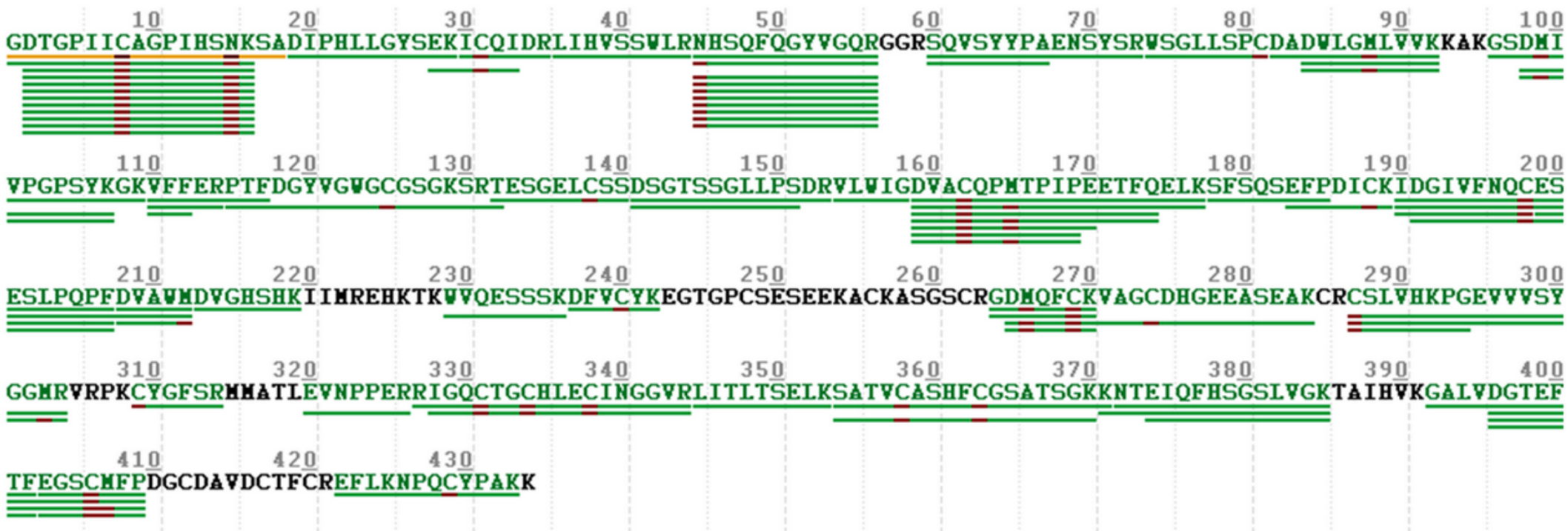


**Supplementary Figure S2. The sequences and the deduced N-glycosylation site of rGn protein.** (A) The amino acid sequence of rGn. SFTSV Gn protein was expressed as a fusion protein with several elements at its N-terminus, including signal peptide ($SS^{Ext}$), his-tag, 10 tandem repeats of Ser-Pro, $(SP)_{10}$ and Tobacco etch virus (TEV) protease cleavage sequences, as highlighted in yellow, green, blue, and grey, respectively. (B) As predicted by NetNGlyc 1.0 program, two potential N-glycosylation consensus sequences (i.e. NKS, NHS, marked in red in panel A) were predicted. (C) Characterization of the rGn protein by LC-MS/MS-based analysis. The resulting amino acid sequence covering 84.79 % of rGn (434 residues) was identified and mapped as the green lines beneath the sequences. The glycan profile was further analyzed and shown in Figure 2.

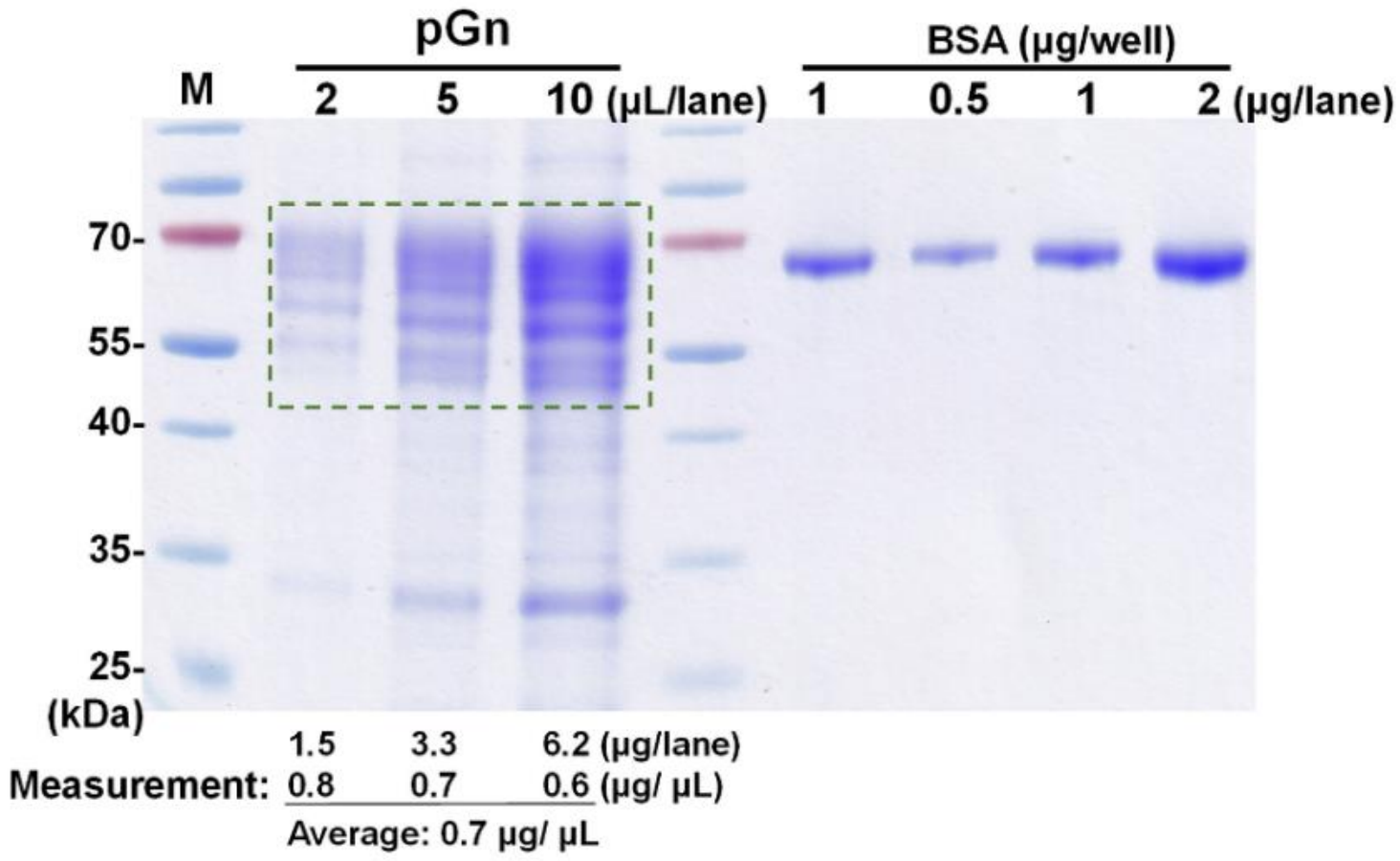


**Supplementary Figure S3. The yield of rGn expressed in plants.** The concentration and total yield of rGn were determined through several steps. Firstly, the purified rGn protein level was quantified by comparison with known concentrations of BSA. The loading volumes of both rGn and BSA were indicated on the SDS-PAGE. To estimate the amount of rGn loaded per lane (μg/lane), a standard curve using BSA was initially established. Additionally, the concentration of rGn was converted to μg per μL (i.e., 0.7 μg/μL). Considering that the volume of purified protein was approximately 1000 μL from 100g of leaves, the total yield of rGn protein is estimated to be approximately 7 mg/kg of leaves. The green boxes indicate rGn proteins selected for quantification.
